# Supplementary material for: Engineering glycosyltransferases into glycan binding proteins using a mammalian surface display platform
Source: Nat Commun. 2025 Jul 18;16:6637. doi: 10.1038/s41467-025-62018-z (PMC12274613; doi:10.1038/s41467-025-62018-z)
Supplement: Supplementary file 1 — Supplementary Information [file 41467_2025_62018_MOESM1_ESM.pdf]

<u>Supplementary Information</u>

Engineering glycosyltransferases into glycan binding proteins using a mammalian surface display platform

Hombu *et al.*

Supplementary Figure 1-12

Supplementary Data 1-4 (in separate .xlsx file)

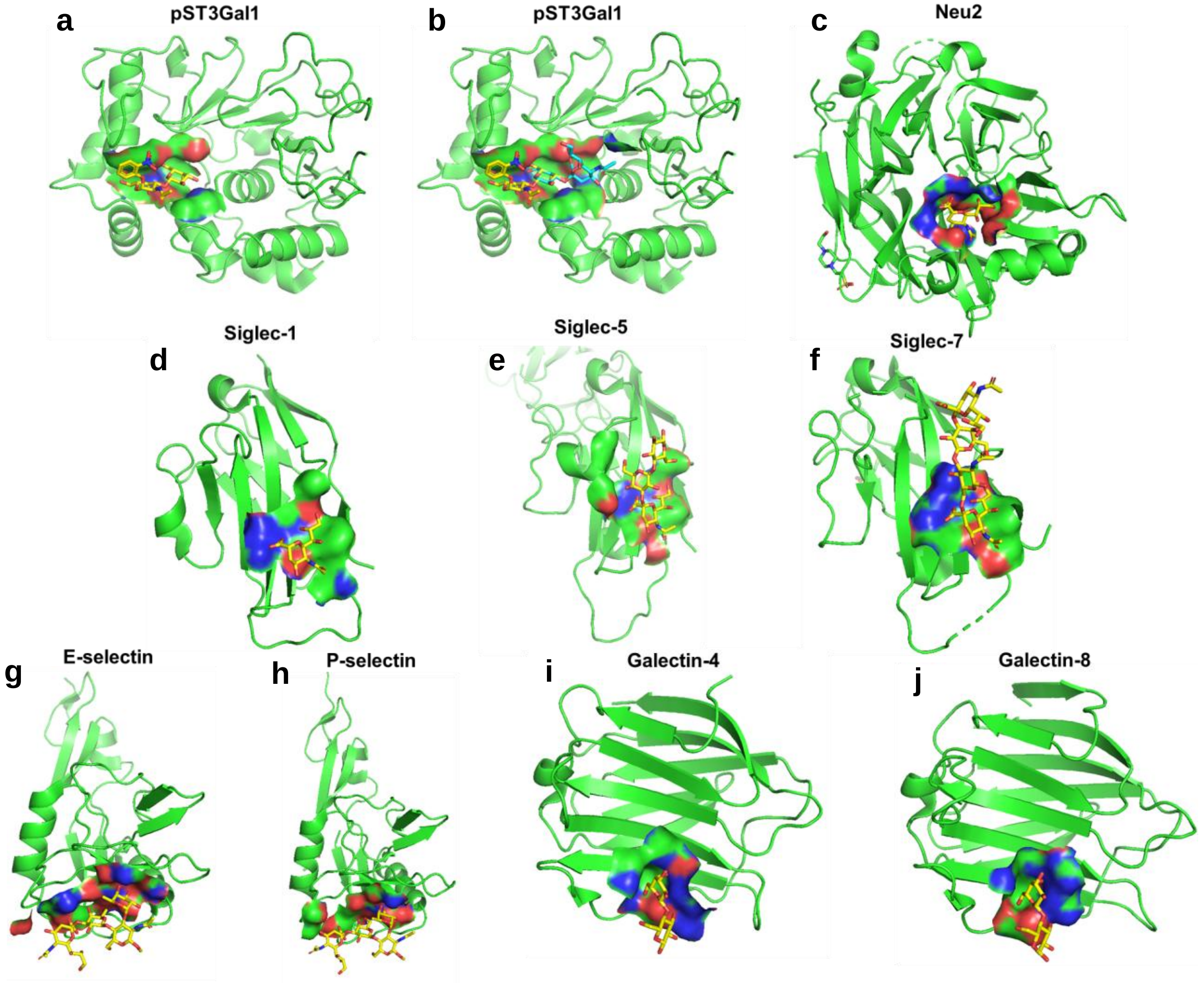

Supple. Fig. 1	Protein name	Host	Protein class	PDB code	Glycan ligand used for defining binding interface	Molecular surface area of binding interface (Å^2)
a	ST3Gal1	Pig	Glycosyltransferase	2WNB	Galβ1,3GalNAcα- (TF antigen)	403.216
b					Galβ1,3GalNAcα- overlayed with Neu5Acα2,3Galβ- (PDB: 5FRE)	541.947
c	Neu2	Human	Glycosidase	1VCU	*N*-acetyl-2,3-dehydro-2-Deoxyneuraminic acid (DANA)	502.476
d	Siglec-1	Mouse	Lectin	1URL	Neu5Acα-	283.675
e	Siglec-5	Human		2ZG3	Neu5Acα2,3Galβ1,3GlcNAcβ-	328.568
f	Siglec-7	Human		2DF3	Neu5Acα2,3Galβ1,4Glcβ-	301.525
g	E-selectin	Human		1G1T	Neu5Acα2,3Galβ1,4[Fucα1,3]GlcNAcβ- (sLe^x)	401.756
h	P-selectin	Human		1G1R	Neu5Acα2,3Galβ1,4[Fucα1,3]GlcNAcβ- (sLe^x)	325.139
i	Galectin-4	Human		5DUV	Galβ1,4Glcβ-	309.455
j	Galectin-8	Human		3AP4	Galβ1,4Glcα-	330.462

Supplementary Fig. 1. Large binding interface of glycosyltransferases. Cartoon representation of glycan-related proteins co-crystalized with glycan ligands and molecular surface area calculation of binding interface between proteins and glycan ligands. Ligands used for defining binding interfaces were represented as yellow sticks, except for overlayed sialoglycan in panel **b** with blue sticks. Whole protein was shown as green cartoons. Binding interfaces were shown as surface. Since pST3Gal1 possesses an undetermined disordered chain that covers bound glycans, our calculations likely under-estimate the binding surface area of pST3Gal1. Although Neu2 also had a deep binding pocket for DANA, the pocket is optimized to recognize the monosaccharide rather than longer sugar chains. Table summarizes the measured surface area/ binding interface between the depicted mammalian proteins and their glycan ligands.

a

Fc-pST3Gal1 WT (PS1)

MIPARFAGVLLALALILPGTLCTGHHHHHHQATEYEYLDYDFLPETEPPRPMMDDDDKSRTCPPCPAPELAGAPSVFLFPP
KPKDTLMISRTPEVTCVVVDVSHEDPEVKFNWYVDGVEVHNAKTKPREEQYNSTYRVVSVLTVLHQDWLNGKEYKCKVS
NKALPAPIEKTISKAKGQPREPQVYTLPPSRDELTKNQVSLTCLVKGFYPSDIAVEWESNGQPENNYKTTPPVLDSDGSFF
LYSKLTVDKSRWQQGNVFSCSVMHEALHNHYTQKSLSLSPGKGSGSGSGSGSRPCTCTRCIEEQRVSAWFDERFNRSM
QPLLTAKNAHLEEDTYKWWLRLQREKQPNNLNDTIRELFQVVPGNVDPLLEKRLVSCRRCAVVGNSGNLKESYYGPQIDS
HDFVLRMNKAPTEGFEADVGSKTTHHFVYPESFRELAQEVSMILVPFKTTDLEWVISATTTGRISHTYVPVPAKIKVKKEKIL
IYHPAFIKYVFDRWLQGHGRYPSTGILSVIFSLHICDEVDLYGFGADSKGNWHHYWENNPSAGAFRKTGVHDGDFESNVT
TILASINKIRIFKGR

b

Fc-CBM40

MIPARFAGVLLALALILPGTLCTGHHHHHHQATEYEYLDYDFLPETEPPRPMMDDDDKSRTCPPCPAPELAGAPSVFLFPP
KPKDTLMISRTPEVTCVVVDVSHEDPEVKFNWYVDGVEVHNAKTKPREEQYNSTYRVVSVLTVLHQDWLNGKEYKCKVS
NKALPAPIEKTISKAKGQPREPQVYTLPPSRDELTKNQVSLTCLVKGFYPSDIAVEWESNGQPENNYKTTPPVLDSDGSFF
LYSKLTVDKSRWQQGNVFSCSVMHEALHNHYTQKSLSLSPGKGSGSGSGSGSLSSLGEYKDINLESSNASNITYDLEKYK
NLDEGTIVVRFNSKDSKIQSLLGISNSKTKNGYFNFYVTNSRVGFELRNQKNEGNTQNGTENLVHMYKDVALNDGDNTVA
LKIEKNKGYKLFLNGKMIKEVKDTNTKFLNNIENLDSAFIGKTNRYGQSNEYNFKGNIGFMNIYNEPLGDDYLLSKTGETK

c

Fc-diCBM40

MIPARFAGVLLALALILPGTLCTGHHHHHHQATEYEYLDYDFLPETEPPRPMMDDDDKSRTCPPCPAPELAGAPSVFLFPP
KPKDTLMISRTPEVTCVVVDVSHEDPEVKFNWYVDGVEVHNAKTKPREEQYNSTYRVVSVLTVLHQDWLNGKEYKCKVS
NKALPAPIEKTISKAKGQPREPQVYTLPPSRDELTKNQVSLTCLVKGFYPSDIAVEWESNGQPENNYKTTPPVLDSDGSFF
LYSKLTVDKSRWQQGNVFSCSVMHEALHNHYTQKSLSLSPGKGSGSGSGSGSLSSLGEYKDINLESSNASNITYDLEKYK
NLDEGTIVVRFNSKDSKIQSLLGISNSKTKNGYFNFYVTNSRVGFELRNQKNEGNTQNGTENLVHMYKDVALNDGDNTVA
LKIEKNKGYKLFLNGKMIKEVKDTNTKFLNNIENLDSAFIGKTNRYGQSNEYNFKGNIGFMNIYNEPLGDDYLLSKTGETKAL
NGSELGSGSGLSSLGEYKDINLESSNASNITYDLEKYKNLDEGTIVVRFNSKDSKIQSLLGISNSKTKNGYFNFYVTNSRVG
FELRNQKNEGNTQNGTENLVHMYKDVALNDGDNTVALKIEKNKGYKLFLNGKMIKEVKDTNTKFLNNIENLDSAFIGKTNR
YGQSNEYNFKGNIGFMNIYNEPLGDDYLLSKTGETK

d

TM(DPP4)-Fc-pST3Gal1 WT (TM-PS1)

MKTPWKTGVLLGLLGAAALVTIITVPVVLLSRTCPPCPAPELAGAPSVFLFPPKPKDTLMISRTPEVTCVVVDVSHEDPEVK
FNWYVDGVEVHNAKTKPREEQYNSTYRVVSVLTVLHQDWLNGKEYKCKVSNKALPAPIEKTISKAKGQPREPQVYTLPPS
RDELTKNQVSLTCLVKGFYPSDIAVEWESNGQPENNYKTTPPVLDSDGSFFLYSKLTVDKSRWQQGNVFSCSVMHEALH
NHYTQKSLSLSPGKGSGSGSGSGSRPCTCTRCIEEQRVSAWFDERFNRSMQPLLTAKNAHLEEDTYKWWLRLQREKQP
NNLNDTIRELFQVVPGNVDPLLEKRLVSCRRCAVVGNSGNLKESYYGPQIDSHDFVLRMNKAPTEGFEADVGSKTTHHFV
YPESFRELAQEVSMILVPFKTTDLEWVISATTTGRISHTYVPVPAKIKVKKEKILIYHPAFIKYVFDRWLQGHGRYPSTGILSVI
FSLHICDEVDLYGFGADSKGNWHHYWENNPSAGAFRKTGVHDGDFESNVTTILASINKIRIFKGR

Supplementary Fig. 2. Fc-protein sequence for Fc-pST3Gal1 WT (PS1), Fc-CBM40, Fc-diCBM40, and cell-surface displayed Fc-pST3Gal1 WT (TM-PS1). (**a**) the VWF signal peptide (yellow), 6xhis tag (blue), 19 amino acids of PSGL-1 (green), enterokinase cleavage site (plain) and human Fc (gray) preceded the target protein. Truncated pST3Gal1 (Δ59) appears in magenta in panel **a**. Amino acids substituted in H302A and Dead (Q108A/Y233A/Y269F) are shown using underlined gray and yellow letters, respectively. (**b**)-(**c**)**.** CBM40 sequence in single (panel b) or concatenated form (panel c) are highlighted in red. (**d**) In TM-PS1, the DPP4 cytoplasmic tail (yellow) and transmembrane region (green) precede human Fc (gray) and truncated pST3Gal1 wild-type (Δ59, magenta). Amino acids mutated in Lib1 and Lib2 are shown using yellow and gray underlined fonts (within magenta highlights).

Hombu *et al.*
Sup. Figure 3

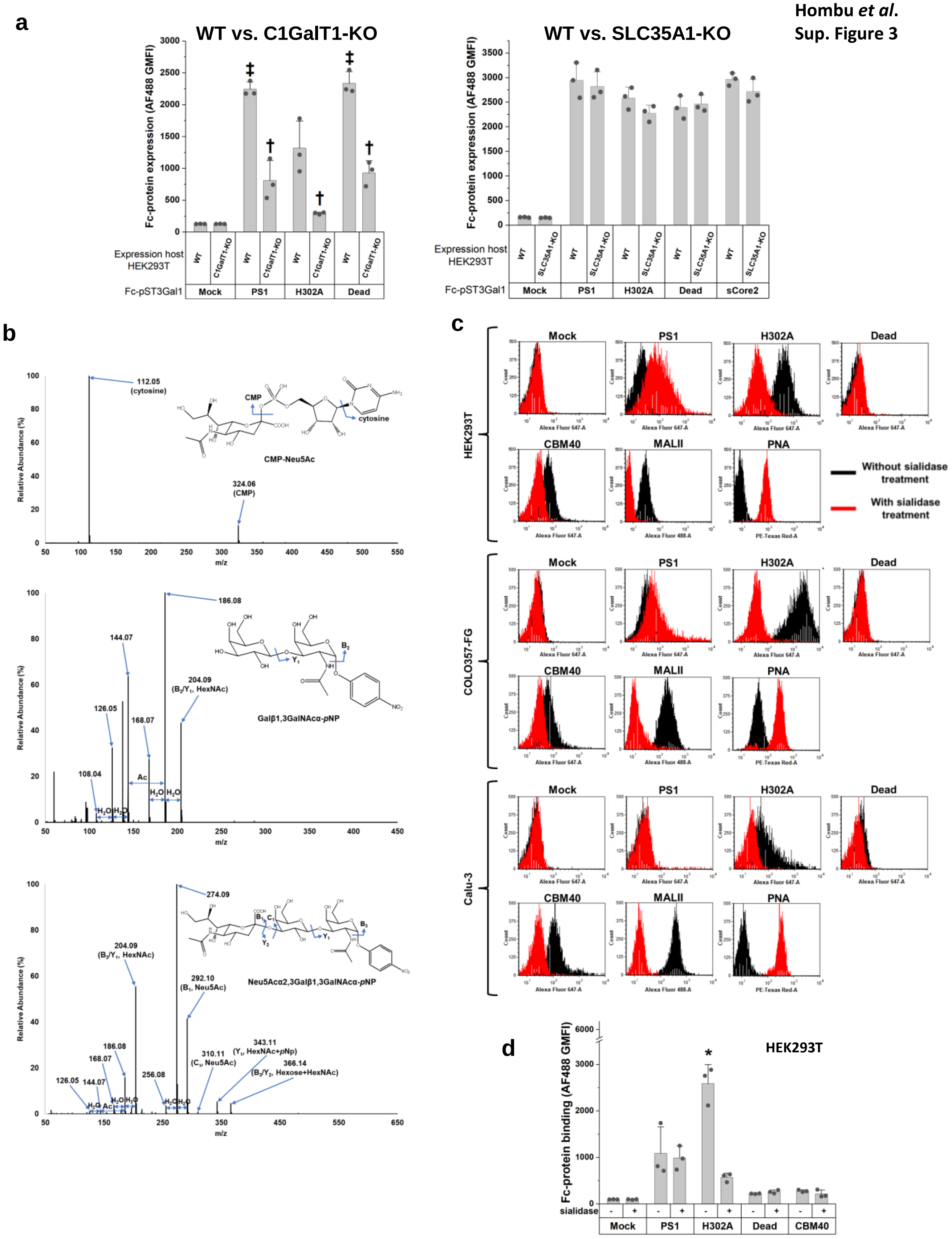

Supplementary Fig. 3. Fc-protein expression, enzyme activity and sialic acid dependence (**a**) Expression of PS1 and its variants in HEK293T WT (wild-type) was compared with respect to protein expression in *C1GalT1*-KO (left panel) and *SLC35A1*-KO (right panel). Flow cytometry FLICA assay was used to quantify Fc-fusion protein production in culture supernatant. (**b**) MS/MS spectra of donor (CMP-Neu5Ac, top), acceptor substrate (Galβ1,3GalNAcα-*p*NP, middle), and product (Neu5Acα2,3Galβ1,3GalNAcα-*p*NP, bottom). (**c**) Flow cytometry measured the binding of fluorescent lectins or Fc-fusion proteins (pre-complexed with fluorescent anti-human Fc specific IgG) to HEK293T, COLO357-FG, or Calu-3 cells, in the presence or absence of sialidase. (**d**) While studies in panel c used AF647 conjugated anti-human Fc, similar studies were performed with AF488-conjugated secondary Ab for reconfirmation. Strong sialic acid dependent binding was observed for H302A using both secondary Abs. Data are mean ± STD (N=3, biological replicates using cells cultured in different wells or dishes). *P*-values were calculated using one-way ANOVA followed by the Tukey post-test (see Source Data file). * $p < 0.05$ with respect to all other samples. ‡$p<0.05$ with respect to all other samples except that samples marked by ‡ are not significantly different from each other; †$p<0.05$ with respect to the same Fc-pST3Gal1 variant samples expressed with HEK293T WT cells.

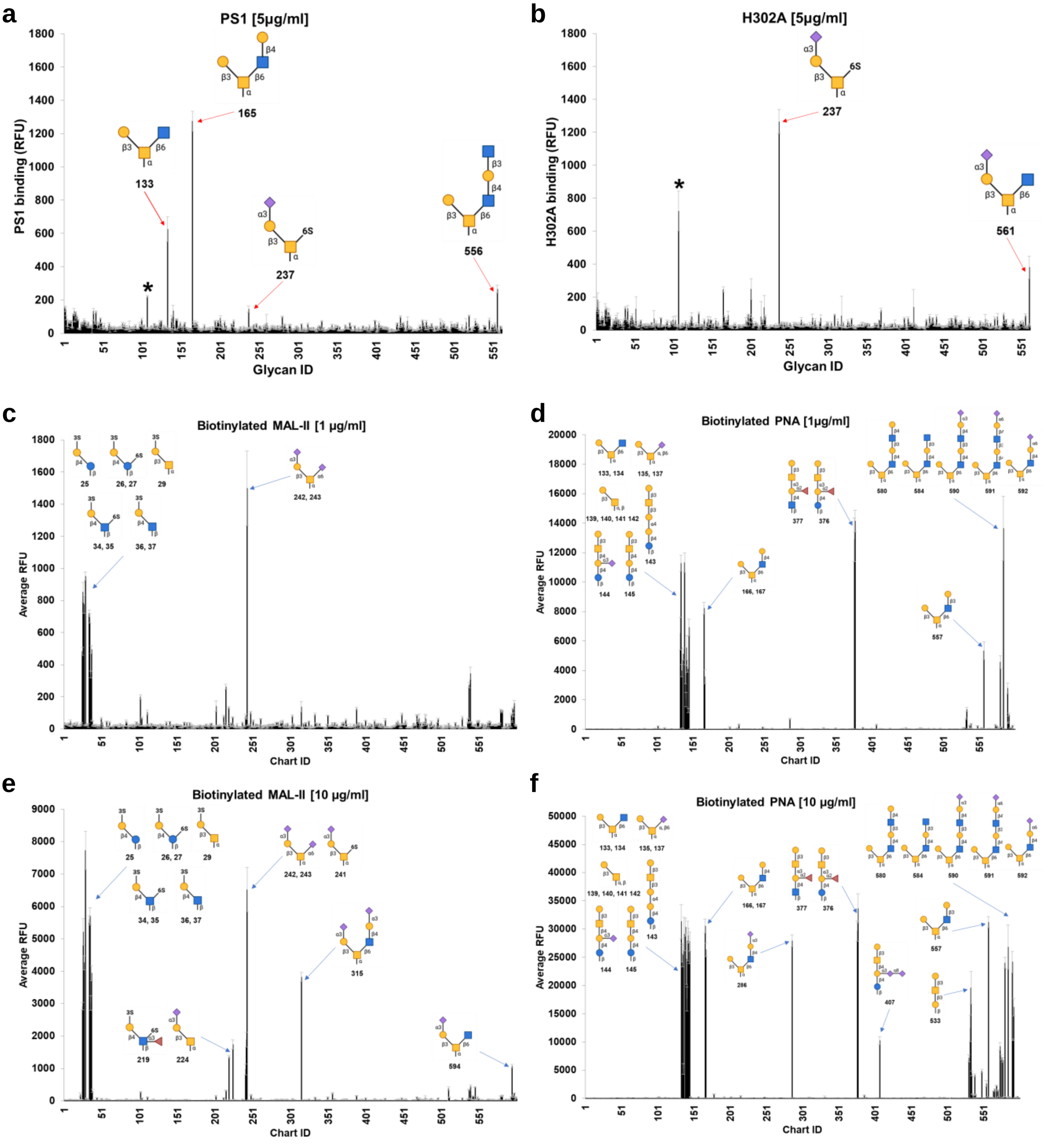

Supplementary Fig. 4. CFG microarray binding data for 5 µg/mL Fc-fusion proteins, MALII, and PNA. (**a**)-(**b**). Lectin binding quantified using relative fluorescence units (RFU) for 5 µg/mL PS1 (panel **a**) and H302A (panel **b**). Data are mean + STD (N=6 using six different microarrays). Red arrows with glycan symbol and number represent strong binders. * Glycan 107 exhibits non-specific binding as its signal does not increase upon increasing Fc-protein concentrations. (**c**)-(**f**). Glycan microarray analysis of biotinylated MALII (panels **c**, **e** at two concentrations) and PNA (panels **d**, **f**) using CFG glycan microarray (*Data source*: National Center for Functional Glycomics (NCFG) website). Strong binders, detected using AF488-conjugated streptavidin, are marked by blue arrows. Binding properties of PS1 and H302A are vastly different from the commercial lectins. Note that different glycan microarrays were used for **a**-**b** vs. **c**-**f**, and thus glycans and chart ID values do not necessarily match. Source data are included in Source Data file.

Hombu *et al*.
Sup. Figure 5

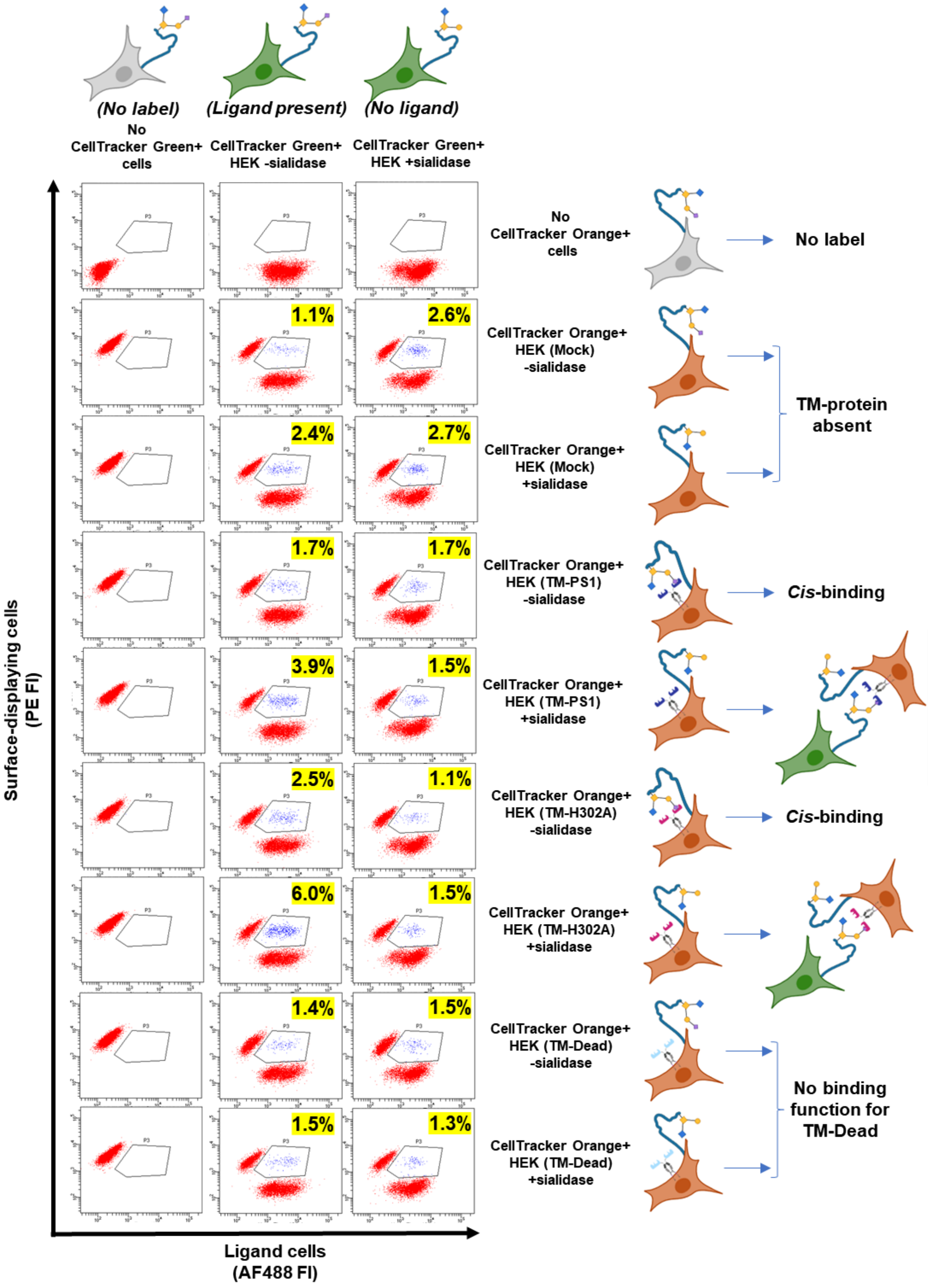

Supplementary Fig. 5. Heterotypic cell binding between ligand and surface-display cells. HEK293T WT cells were labeled with CellTracker Green, and either sialidase treated (third column) or not treated (second column). HEK293T WT cells were transfected with TM-PS1, TM-H302A, or TM-Dead and labeled with CellTracker Orange (along rows). These were either sialidase treated or not treated. Upon mixing the same number of two cell populations for 20 min at RT, heterotypic green-orange cell aggregates were only observed upon mixing HEK cells without sialidase (labeled green) with HEK cells bearing TM-PS1 or TM-H302A that were sialidase treated. In the case of TM-PS1 background 1.7% binding was increased to 3.9% upon sialidase treatment. For TM-H302A binding increased from 2.5% (without sialidase) to 6% (with sialidase). In negative controls, neither TM-Dead nor Mock transfected cells displayed such increase. Additionally, removing sialic acid from HEK293T/green cells reduced heterotypic cellular interactions to 1.5% (baseline). Thus, *cis*-interaction occur between TM-PS1/H302A and host sialoglycans. This prevents heterotypic cell interactions unless cells are sialidase treated. Similar to this, *cis*-interactions may prevent surface-displayed Fc-proteins from binding the sialyl core-2 PAA-FITC probe. This is prevented by expressing surface display proteins on *SLC35A1*-KO cells that lack sialic acids. Created in BioRender. Neelamegham, S. (2025) https://BioRender.com/tigz92o

Hombu *et al.*
Sup. Figure 6

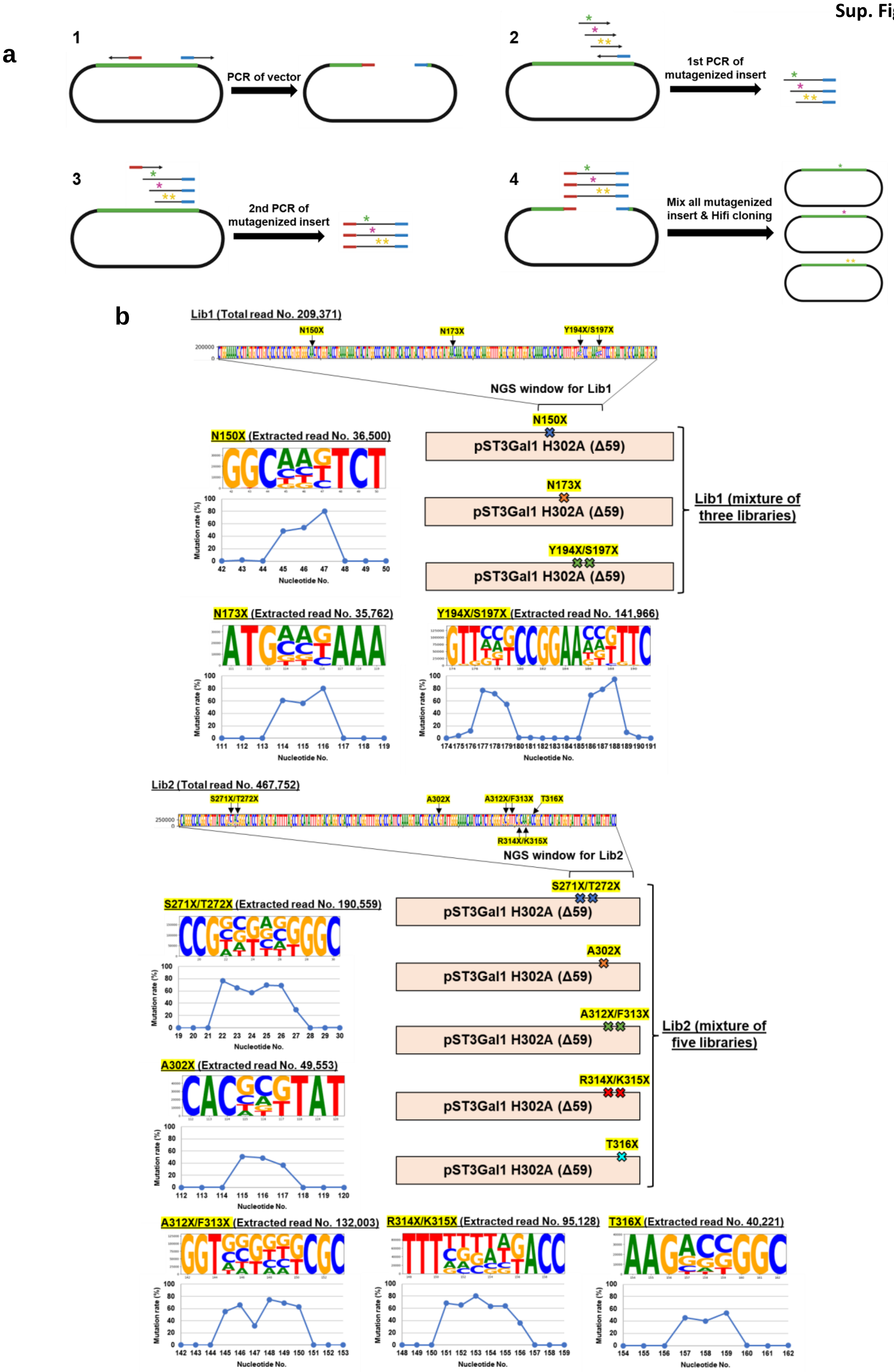

Supplementary Fig. 6. Lectin surface display library construction. (**a**) Cloning strategy for constructing Lib1 and Lib2. Gene of interest is shown in green. Vector was first linearized using PCR primers that flank the insert site (red and blue). Two-step PCR was performed to introduce mutations. In the 1st PCR, the forward primer contained NNK mutations (shown as green, pink, yellow stars) while the reverse primer overlapped with the linearized plasmid. This product was used as a megaprimer in the 2nd PCR along with a forward primer that overlapped with the linearized vector. The final PCR product was then ligated with the linearized vector using Hifi cloning. As all mutagenized primers have 1-2 NNK sites, resulting plasmid is the mixture of plasmids with 1 or 2 mutations. (**b**) Mutation profiles of Lib1 and Lib2. The amplicon was generated from the mutagenized plasmids and subjected to NGS (next-generation sequencing). Nucleotides corresponding from L135 to S206 were included in the amplicon of Lib1, and ones from L263 and T328 were in the amplicon of Lib2. Each nucleotide (A, T, C, and G) was shown in green, red, blue, and yellow, respectively. Mutagenized residues were pointed with arrows and yellow highlights. Because Lib1 and Lib2 are the mixture of plasmids with one or two mutations, each component was extracted in NGS read processing by fixing the mutagenized nucleotides to the original nucleotide sequences except for the mutations of interest. Logos and mutation rates of extracted reads were shown for each mutation. Source data are part of Source Data file. Created in BioRender. Neelamegham, S. (2025) https://BioRender.com/tigz92o

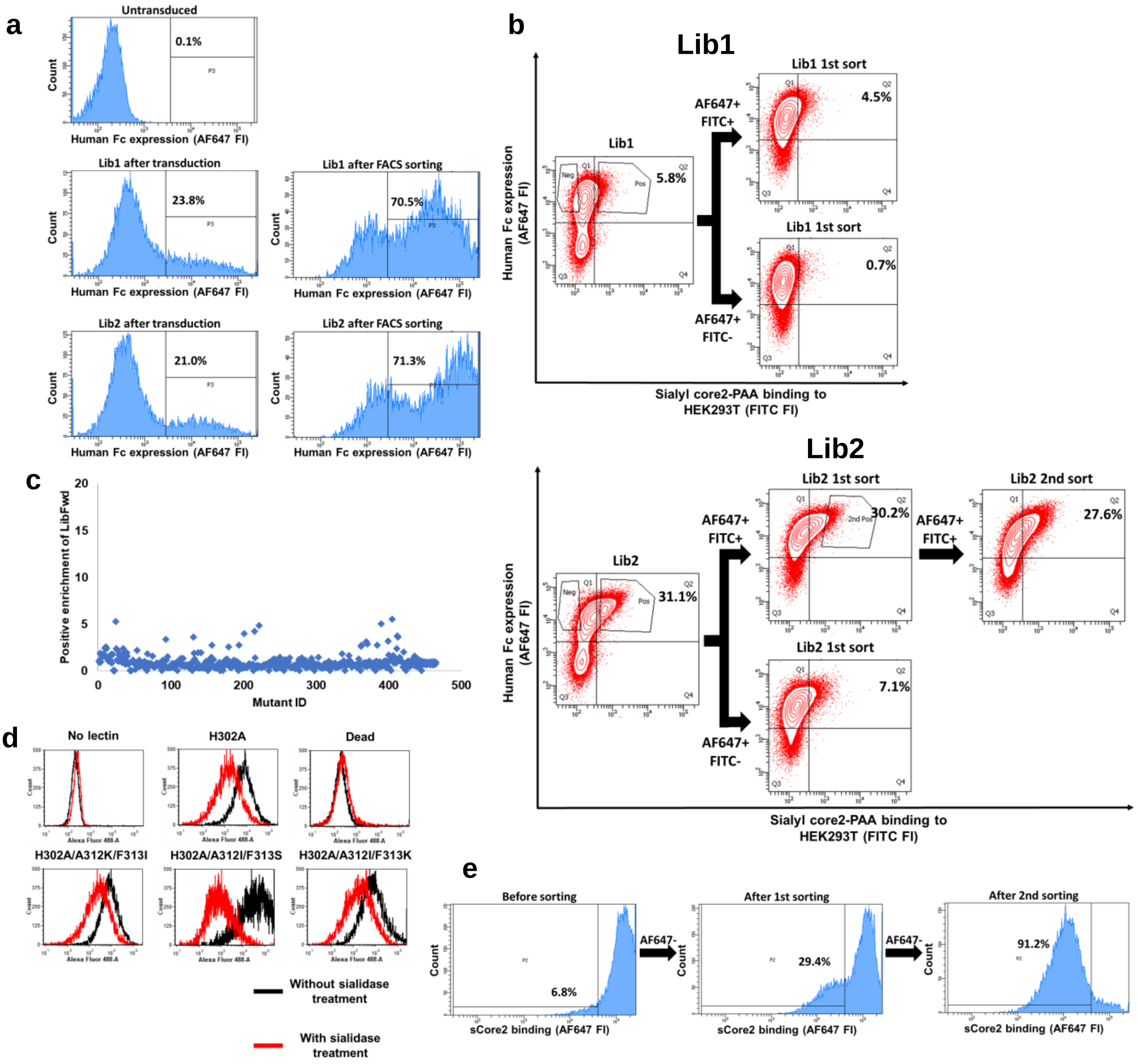

Supplementary Fig. 7. Construction, sorting, and characterization of selected mutants using TM-H302A Lib1 and Lib2. (**a**) Lentivirus transduction and FACS sorting of HEK293T Lib1 and Lib2. Histogram demonstrates that less than 30% of the cell population was lentivirally transduced ensuring M.O.I. (multiplicity of infection) < 1. FACS sort enrichment of transduced cells was performed based on cell-surface Fc-protein detection. (**b**) FACS sorting strategy using sialyl-core 2 PAA-FITC for HEK293T Lib1 and Lib2. Sorting gates were shown in contour plots of Lib1 1st sort and Lib2 1st and 2nd sorts based on binding of Fc positive cells to sialyl core-2-PAA. Also shown is the negative sort which encompasses the non-binders. Percentage of quadrant 2 (Q2) was shown for each plot. (**c**) Positive enrichment scores of mutants in Lib1. Only one positive sort was performed for Lib1 as the binding of sialyl core-2-PAA to these cells was low, whereas two positive sorts were done for Lib2. (**d**) Sialidase dependence of selected mutants. Flow cytometry histograms show binding of purified Fc-proteins, pre-complexed with AF488-conjugated anti-human Fc specific IgG to HEK293T cells treated with or without sialidase. Data are representative of triplicate runs. (**e**) FACS sorting strategy for HL60 glycoCRISPR library using sCore2. Sorting gates for negative binders were shown in histograms of Before sorting and After 1st sorting based on sCore2 binding. Percentage of negative populations was shown for each plot. Source data are provided in Source Data file.

a

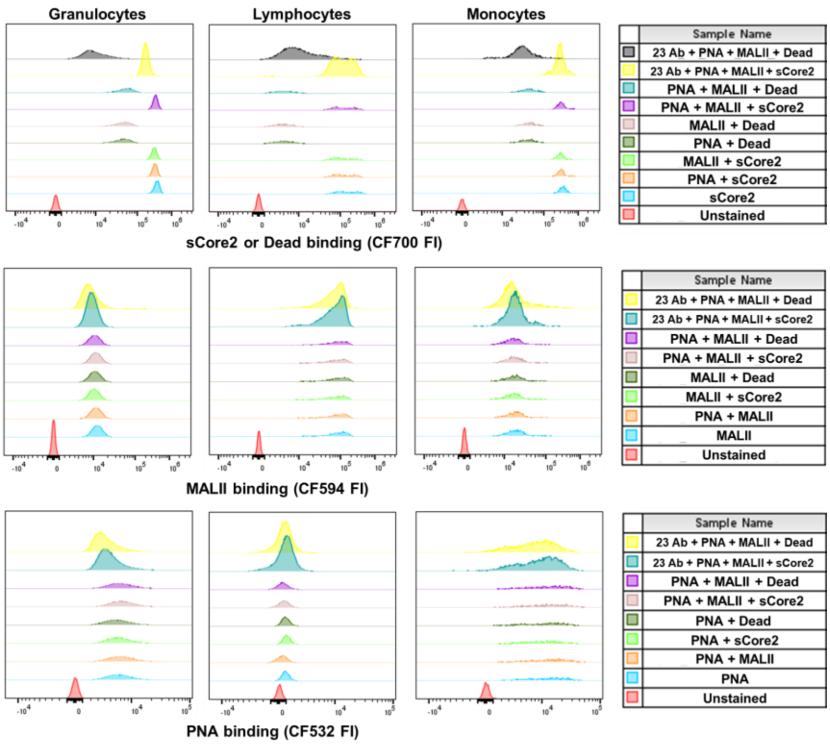
Granulocytes
Lymphocytes
Monocytes
Sample Name
23 Ab + PNA + MALII + Dead
23 Ab + PNA + MALII + sCore2
PNA + MALII + Dead
PNA + MALII + sCore2
MALII + Dead
PNA + Dead
MALII + sCore2
PNA + sCore2
sCore2
Unstained
sCore2 or Dead binding (CF700 FI)
PNA + MALII
MALII
MALII binding (CF594 FI)
PNA
PNA binding (CF532 FI)

b

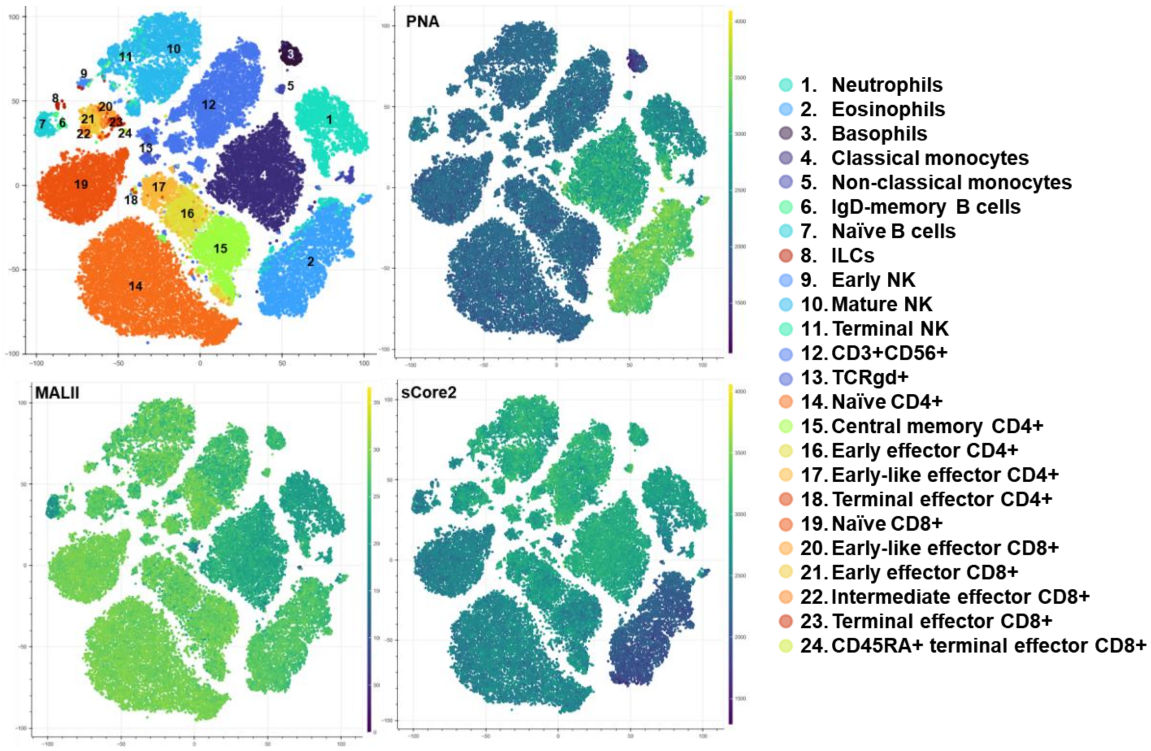
PNA
MALII
sCore2
1. Neutrophils
2. Eosinophils
3. Basophils
4. Classical monocytes
5. Non-classical monocytes
6. IgD-memory B cells
7. Naïve B cells
8. ILCs
9. Early NK
10. Mature NK
11. Terminal NK
12. CD3+CD56+
13. TCRgd+
14. Naïve CD4+
15. Central memory CD4+
16. Early effector CD4+
17. Early-like effector CD4+
18. Terminal effector CD4+
19. Naïve CD8+
20. Early-like effector CD8+
21. Early effector CD8+
22. Intermediate effector CD8+
23. Terminal effector CD8+
24. CD45RA+ terminal effector CD8+

c

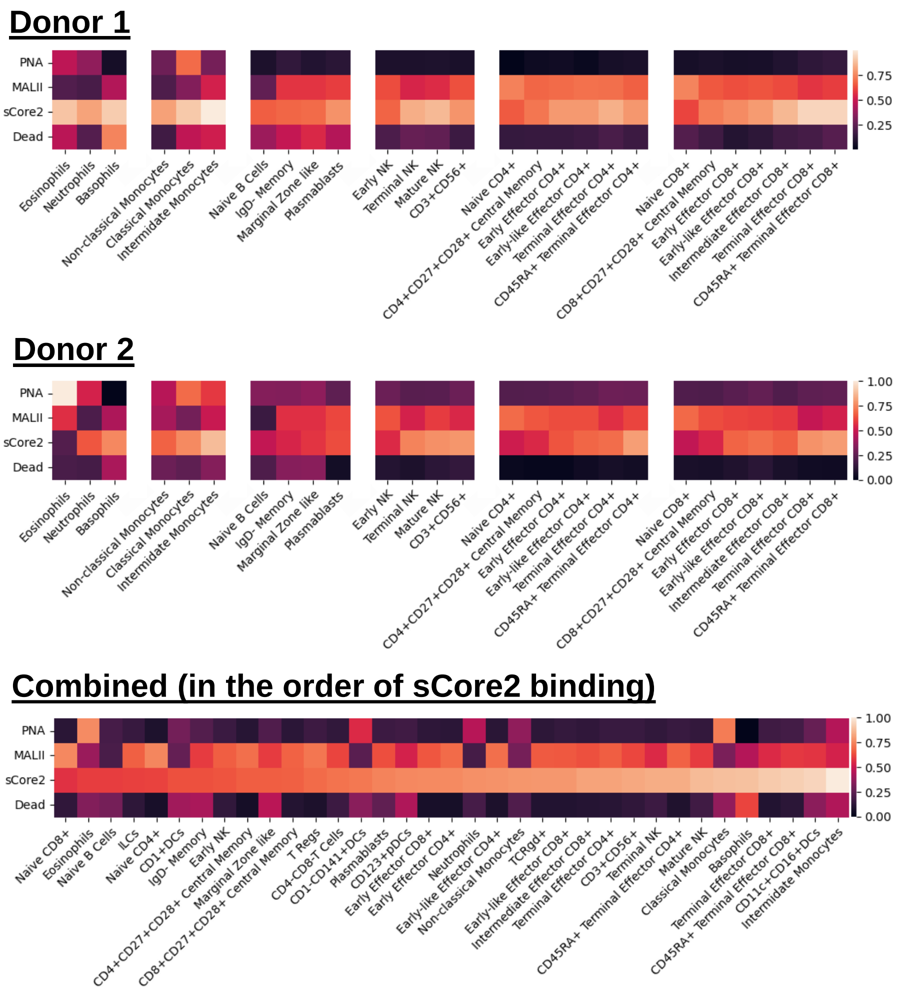

d

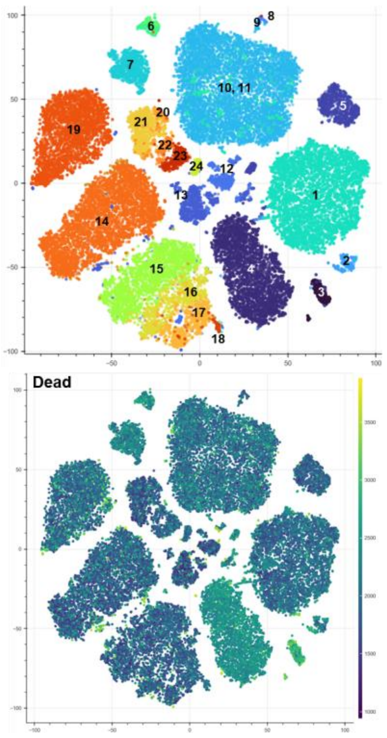

Supplementary Fig. 8. Spectral flow cytometry analysis of sCore2 and lectins. (**a**) Lack of competition between commercial lectins and sCore2. Histograms confirm lack of competition between PNA, MALII and sCore2/Dead in cell binding assay using peripheral blood cells. Histograms are shown for granulocytes, lymphocytes and monocytes based on FSC and SSC gating and for blood donor 1. Note the presence of multiple sCore2 binding populations in the lymphocyte gate. Representative data from donor 1 is shown. (**b**) tSNE plots showing the different binding patterns of PNA, MALII and sCore2 for different human peripheral blood cells. Representative data are shown for blood cells from donor 2, immunoprofiled using 23 antibodies against human antigens. Numbers and colors in legends correspond to individually labeled cell populations in the tSNE plots. PNA bound to myeloid cell types except for basophils, MALII showed higher binding to lymphoid cell types than myeloid cell types, and sCore2 bound to myeloid cell types and terminally differentiated lymphoid cells. (**c**) Heatmaps showing the binding of each lectin for human peripheral blood cells from individual donors. Results from donor 1 only, donor 2 only, and both donors were visualized. The order of heatmaps are aligned based on the cell type (donor 1 or 2 only) or the binding intensity of sCore2 (combined). (**d**) Binding of Dead to peripheral blood cells was low. tSNE plots show low binding of Dead to different human peripheral blood cell types. Source data are provided in Source Data file.

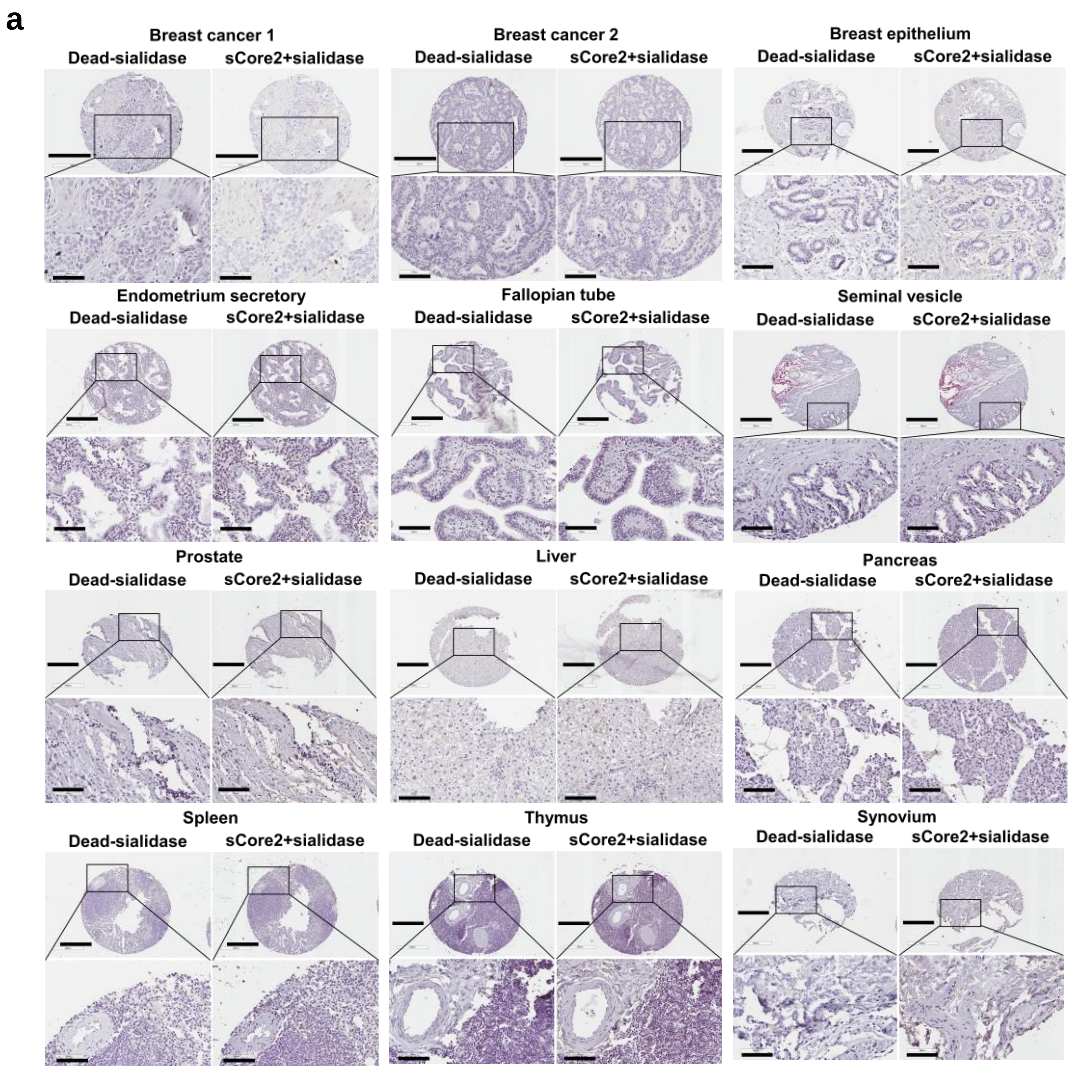

Supplementary Fig. 9 (panel a). Human normal tissue microarray stained using sCore2 or Dead. (**a**) Human normal tissue microarray and breast cancer tissue analysis was performed using Dead or sCore2. Treatment of tissue with sialidase prevented sCore2 binding, while strong sCore2 binding was noted in absence of sialidase in Fig. 6 (main manuscript). Binding using the Dead negative control reagent was also low. Note that red stains in seminal vesicle pre-exists even before deparaffinization. Scale bars = 400 μm for main images (except 300 μm for breast cancer). Scale bars for magnifications = 100 μm.

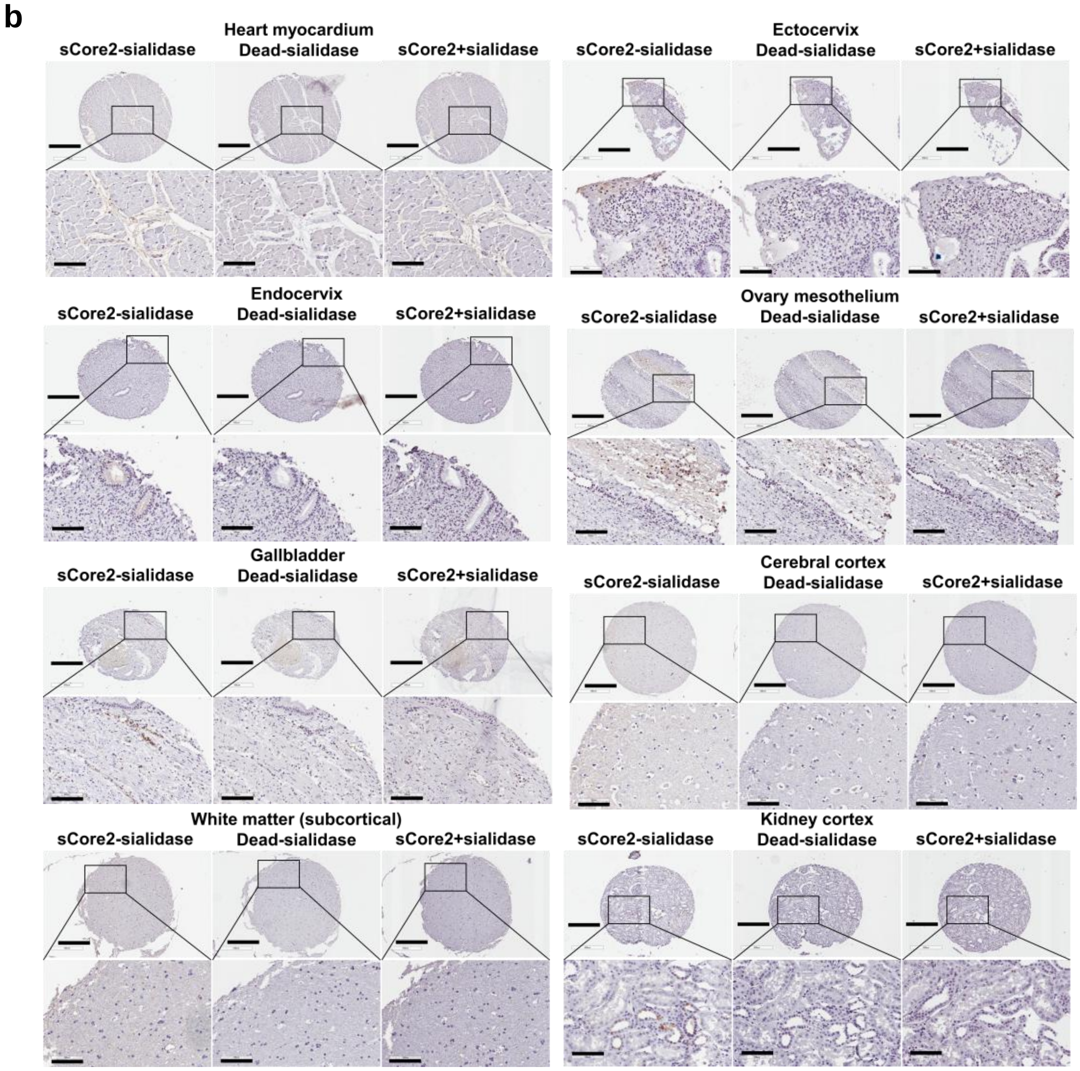

Supplementary Fig. 9 (panel b). Human normal tissue microarray stained using sCore2 or Dead. (**b**) Human normal tissue microarray analysis. Several tissues with moderate staining by sCore2-sialidase were shown individually along with negative controls. Representative images from two independent experiments is shown. Scale bars = 400 μm for main images. Scale bars for magnifications = 100 μm.

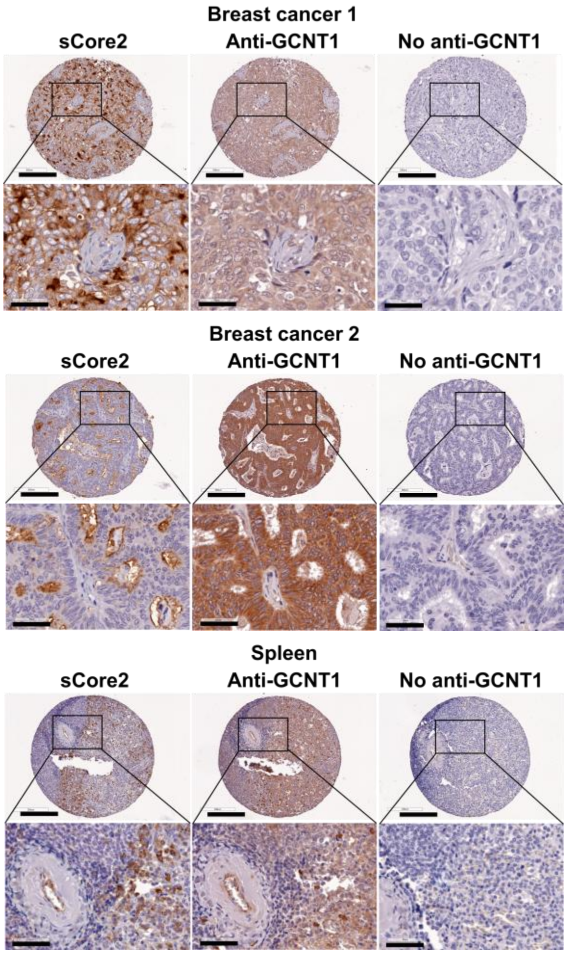

Supplementary Fig. 10. Human tissue staining comparison using sCore2 or anti-GCNT1 antibody. Human breast cancer and normal spleen tissues were stained using sCore2 or anti-GCNT1 antibody. Staining with only secondary antibody but no anti-GCNT1 serves as a negative control. sCore2 stained specific tissue region, whereas anti-GCNT1 stained tissues more broadly. Some overlaps were observed for staining of sCore2 and anti-GCNT1. Representative images from two independent experiments. Main figure scale bars = 200 μm, magnification scale bar = 50 μm.

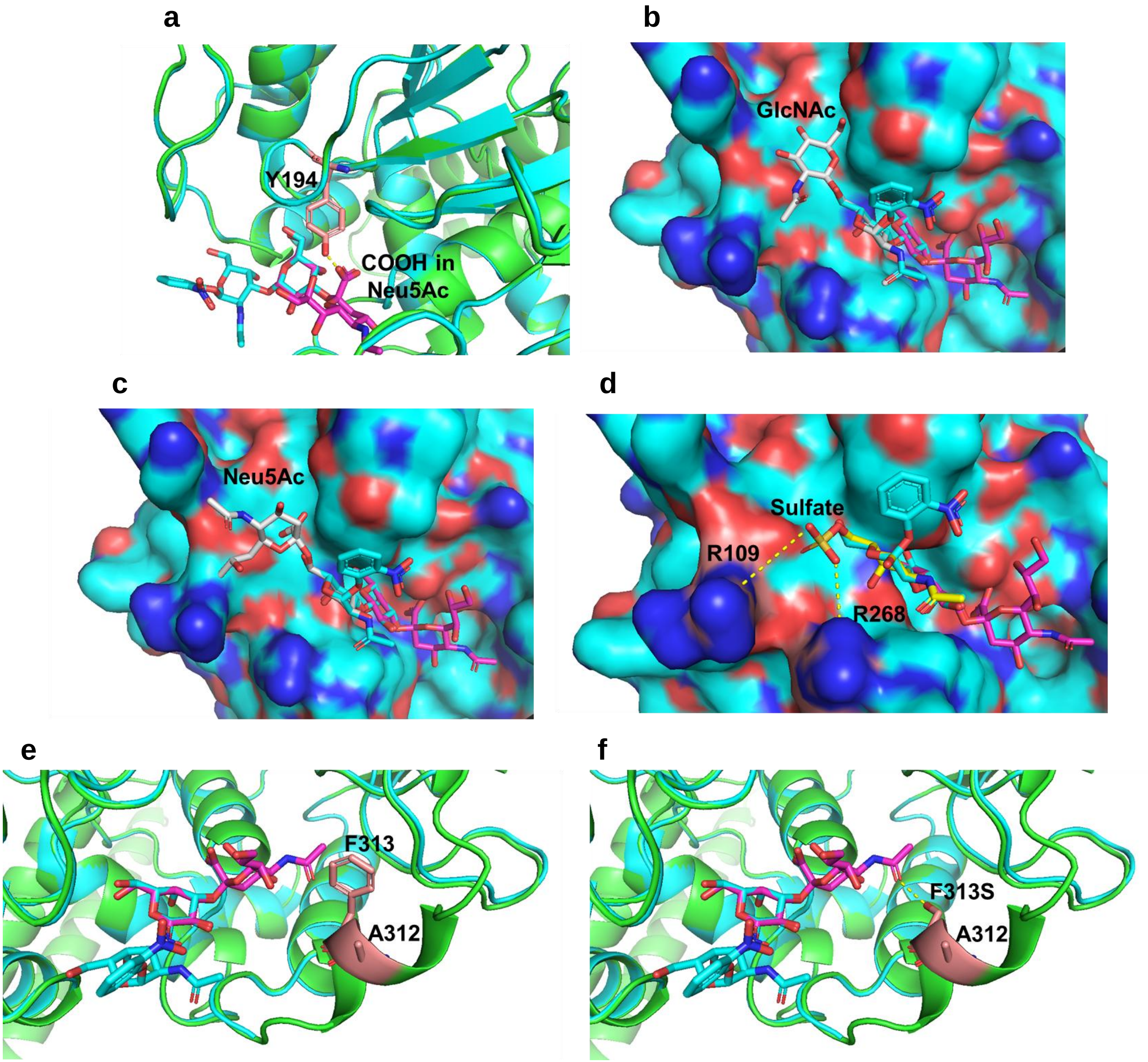

Supplementary Fig. 11. Cartoon representation of pST3Gal1 and effects of specific amino acids, mutations, and overlayed ligands. (**a**) Y194 in pST3Gal1 in proximity to carboxyl group in superimposed Neu5Ac in sialoglycan ligand. (**b**) Superimposed core 2-GlcNAc accepted in the pocket of pST3Gal1. (**c**) Superimposed α2,6-Neu5Ac. Glycerol-like side chain was hidden by pST3Gal1 shown as surface, indicating that steric hinderance may be caused. (**d**) R109 and R268 potentially interacting with sulfate group of sulfated sialyl TF-antigen identified in glycan microarray. These Arginines are considered to be flexible, and so they can be directed towards the proximal sulfate group. (**e**) Unmutated F313 is located near Neu5Ac. (**f**) F313S potentially interacting with C5 acetamido group in Neu5Ac. pST3Gal1 co-crystalized with CMP and Galβ1,3GalNAcα-*p*NP (PDB: 2WNB) and predicted by Alphafold were shown in cyan and green cartoons, respectively. In panels **b**-**d**, crystal structure of pST3Gal1 was shown as surface. Y194, A312, and F313 were shown as orange sticks. Galβ1,3GalNAcα-*p*NP, Neu5Acα2,3Gal superimposed from 5FRE, and (6S)GalNAc superimposed from 6S20 were shown as cyan, magenta, and yellow sticks, respectively. GlcNAcβ1,6GalNAc superimposed from 6K2N and Neu5Acα2,6Gal superimposed from 3PHZ were shown as white sticks. Potential interactions between atoms in residues and ligands are shown as yellow dotted lines. In Fig. **b**-**d**, dihedral angles of several bonds were adjusted so the ligand fits in pST3Gal1 with better conformation.

a

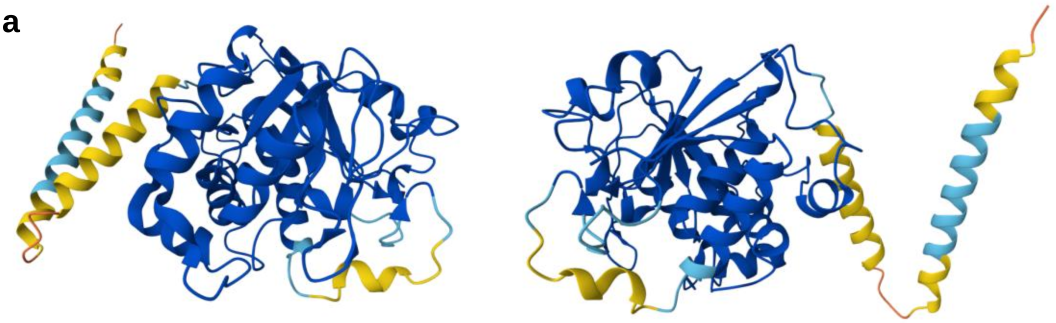

b

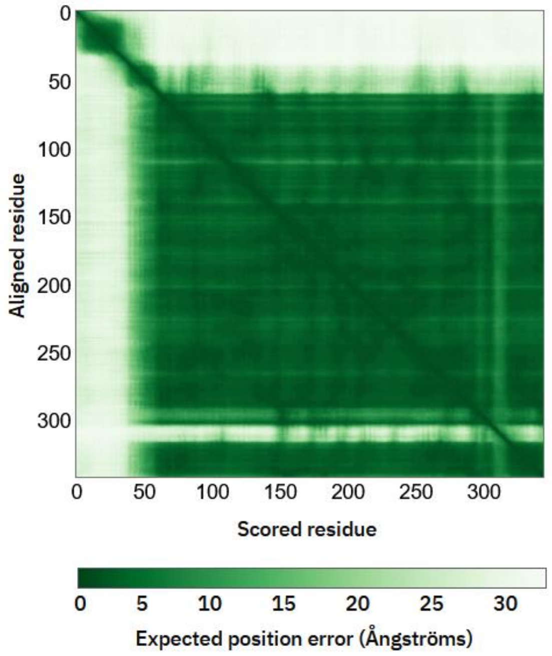

Supplementary Fig. 12. Protein prediction model of pST3Gal1. (**a**) pLDDT scores overlaid on pST3Gal1 structure predicted by AlphaFold (model version: AF-Q02745-F1-v4) from two viewing angles. Residues with very high (pLDDT > 90), high (90 > pLDDT > 70), low (70 > pLDDT > 50), or very low (pLDDT < 50) prediction confidence were colored in blue, light blue, yellow, or orange, respectively. (**b**) Prediction aligned error (PAE) plot of the predicted pST3Gal1 structure.
