# Supplementary material for: Engineering glycosyltransferases into glycan binding proteins using a mammalian surface display platform
Source: Nat Commun. 2025 Jul 18;16:6637. doi: 10.1038/s41467-025-62018-z (PMC12274613; doi:10.1038/s41467-025-62018-z)
Supplement: Supplementary file 2 — Description of Additional Supplementary Files [file 41467_2025_62018_MOESM2_ESM.pdf]

Description of Additional Supplementary Files

File Name: Supplementary Data 1
Description: Glycan microarray data for PS1 and H302A

File Name: Supplementary Data 2
Description: Mutant enrichment score: Data are provided for 1st round (negative and 1st positive samples) and 2nd round (2nd positive sample) sorted samples

File Name: Supplementary Data 3
Description: Details of tissue microarray staining by sCore2

File Name: Supplementary Data 4
Description: Primers used in this study
