# Supplementary material for: Engineering glycosyltransferases into glycan binding proteins using a mammalian surface display platform
Source: Nat Commun. 2025 Jul 18;16:6637. doi: 10.1038/s41467-025-62018-z (PMC12274613; doi:10.1038/s41467-025-62018-z)
Supplement: Supplementary file 4 — Reporting Summary [file 41467_2025_62018_MOESM4_ESM.pdf]

nature portfolio

Corresponding author(s): Neelamegham, Sriram

Last updated by author(s): Jun 16, 2025

Reporting Summary

Nature Portfolio wishes to improve the reproducibility of the work that we publish. This form provides structure for consistency and transparency in reporting. For further information on Nature Portfolio policies, see our Editorial Policies and the Editorial Policy Checklist.

Statistics

For all statistical analyses, confirm that the following items are present in the figure legend, table legend, main text, or Methods section.

n/a	Confirmed	
☐	☒	The exact sample size (*n*) for each experimental group/condition, given as a discrete number and unit of measurement
☐	☒	A statement on whether measurements were taken from distinct samples or whether the same sample was measured repeatedly
☐	☒	The statistical test(s) used AND whether they are one- or two-sided<br>*Only common tests should be described solely by name; describe more complex techniques in the Methods section.*
☐	☒	A description of all covariates tested
☐	☒	A description of any assumptions or corrections, such as tests of normality and adjustment for multiple comparisons
☐	☒	A full description of the statistical parameters including central tendency (e.g. means) or other basic estimates (e.g. regression coefficient) AND variation (e.g. standard deviation) or associated estimates of uncertainty (e.g. confidence intervals)
☐	☒	For null hypothesis testing, the test statistic (e.g. *F*, *t*, *r*) with confidence intervals, effect sizes, degrees of freedom and *P* value noted<br>*Give P values as exact values whenever suitable.*
☒	☐	For Bayesian analysis, information on the choice of priors and Markov chain Monte Carlo settings
☒	☐	For hierarchical and complex designs, identification of the appropriate level for tests and full reporting of outcomes
☒	☐	Estimates of effect sizes (e.g. Cohen's *d*, Pearson's *r*), indicating how they were calculated

Our web collection on statistics for biologists contains articles on many of the points above.

Software and code

Policy information about availability of computer code

Data collection	All sequencing was performed using standard Illumina sequencers.<br>Glycan microarray acquisition and analysis was performed by the National Center for Functional Glycomics resource as described at: https://research.bidmc.org/ncfg/microarrays.<br>Spectral flow cytometry data were collected using Spectroflo v3.2.1 software (Cytek, Inc).<br>BD Fortessa X-20 data were collected using FACSDiva 8.0.1 software.
Data analysis	Forward genetic screen sgRNA quantification was performed using MAGeCK (0.5.9) available at https://sourceforge.net/p/mageck/wiki/Home/.<br>Representation of mutants in pST3Gal1 libraries was quantified using SeqKit (version 2.3.1).<br>Spectral flow cytometry data compensation and gating were performed using FlowJo v9. These data were exported into dataframes (pandas v2.2.2) using Flowkit v1.0.1. Resulting dataframes were normalized and scaled using SciKit-learn v1.1.3. Following PCA and tSNE generation also using Scikit-learn, final results were rendered using Bokeh v3.6.0 (bokeh.org).

For manuscripts utilizing custom algorithms or software that are central to the research but not yet described in published literature, software must be made available to editors and reviewers. We strongly encourage code deposition in a community repository (e.g. GitHub). See the Nature Portfolio guidelines for submitting code & software for further information.

Data

Policy information about availability of data

All manuscripts must include a data availability statement. This statement should provide the following information, where applicable:

- Accession codes, unique identifiers, or web links for publicly available datasets
- A description of any restrictions on data availability
- For clinical datasets or third party data, please ensure that the statement adheres to our policy

All data supporting the results of this study can be found in the article, supplementary, and source data files.. Source Data are also available at Figshare [https://doi.org/10.6084/m9.figshare.28909301], and also provided with this paper. NGS data are deposited as part of NCBI BioProject: PRJNA1173749 [https://www.ncbi.nlm.nih.gov/bioproject/PRJNA1173749/]. Plasmids for Fc-pST3Gal1 WT, H302A, Dead and sCore2 are deposited at Addgene. Any other plasmids described in this paper will be provided by the corresponding author.

Research involving human participants, their data, or biological material

Policy information about studies with human participants or human data. See also policy information about sex, gender (identity/presentation), and sexual orientation and race, ethnicity and racism.

Reporting on sex and gender	The study uses human peripheral blood cells for testing lectins of interest. Two donors of both sexes were used. As the focus was on lectin characterization, detailed analysis based on sex/gender was not performed.
Reporting on race, ethnicity, or other socially relevant groupings	Race is not a factor considered in the study, as we only analyzed a limited cohort of healthy human subjects.
Population characteristics	Healthy adult volunteers, either sexes, age 18-60.
Recruitment	Healthy volunteers were recruited from the local student population, based on postings or word-of-mouth at the University.
Ethics oversight	The human subjects protocols used to obtain peripheral blood was approved by the University at Buffalo, Health Sciences Institutional Review Board (HSIRB).<br>The tissue microarrays containing de-identified human tissue were collected and processed by the Cooperative Human Tissue Network (University of Virginia). This portion of the study was determined to not involve human subjects by the HSIRB.

Note that full information on the approval of the study protocol must also be provided in the manuscript.

Field-specific reporting

Please select the one below that is the best fit for your research. If you are not sure, read the appropriate sections before making your selection.

☒ Life sciences ☐ Behavioural & social sciences ☐ Ecological, evolutionary & environmental sciences

For a reference copy of the document with all sections, see nature.com/documents/nr-reporting-summary-flat.pdf

Life sciences study design

All studies must disclose on these points even when the disclosure is negative.

Sample size	No sample-size calculation was performed.
Data exclusions	No data were excluded. Neutrophils were downsampled in the tSNE plots to reduce the number of corresponding dots and improve visual presentation.
Replication	Number of biological replicates performed for each experiment can be determined using the discrete data points displayed in individual panels and also the source data. The manuscript does not present technical replicates unless explicitly stated. Typically we performed three or more replicates for each experiment as this is needed for statistical tests. Reagents described in this paper were reproduced with multiple batches of the product, and using biological replicates. Sialidase treatment effects were consistently observed with sCore2. Effect of core2 structure on lectin binding was noted using both knockout cells and glycan microarrays. The observations were independently reproduced by two independent investigators in the laboratory.
Randomization	Randomization is not relevant as this is a basic science investigation. There are no treatment and test groups.
Blinding	Blinding is not necessary as this is a basic science investigation, reproduced by two operators in the laboratory.

Reporting for specific materials, systems and methods

We require information from authors about some types of materials, experimental systems and methods used in many studies. Here, indicate whether each material, system or method listed is relevant to your study. If you are not sure if a list item applies to your research, read the appropriate section before selecting a response.

Materials & experimental systems

n/a	Involved in the study
☐	☒ Antibodies
☐	☒ Eukaryotic cell lines
☒	☐ Palaeontology and archaeology
☒	☐ Animals and other organisms
☒	☐ Clinical data
☒	☐ Dual use research of concern
☒	☐ Plants

Methods

n/a	Involved in the study
☒	☐ ChIP-seq
☐	☒ Flow cytometry
☒	☐ MRI-based neuroimaging

Antibodies

Antibodies used: All antibody and lectin suppliers, clone names (where applicable) and catalog numbers are provided in main manuscript.

Validation: Primary antibodies from BD Biosciences, Biolegend, and Cytek Biosciences were validated based on manufacture's website. Their binding to specific cell types in spectral flow cytometry studies was as expected. Binding specificity of secondary antibodies were validated using negative controls described in the manuscript.

Eukaryotic cell lines

Policy information about cell lines and Sex and Gender in Research

Cell line source(s): HEK293T and Calu-3 cells were from ATCC. COLO357-FG cells were from Dr. Moorthy Ponnusamy (University of Nebraska Medical College).

Authentication: Cells were not authenticated as the purpose of the study was on lectin binding in basic science context, rather than functional response of cells in cell biology assays.

Mycoplasma contamination: Cell lines were mycoplasma negative

Commonly misidentified lines (See ICLAC register): None

Plants

Seed stocks: *Report on the source of all seed stocks or other plant material used. If applicable, state the seed stock centre and catalogue number. If plant specimens were collected from the field, describe the collection location, date and sampling procedures.*

Novel plant genotypes: *Describe the methods by which all novel plant genotypes were produced. This includes those generated by transgenic approaches, gene editing, chemical/radiation-based mutagenesis and hybridization. For transgenic lines, describe the transformation method, the number of independent lines analyzed and the generation upon which experiments were performed. For gene-edited lines, describe the editor used, the endogenous sequence targeted for editing, the targeting guide RNA sequence (if applicable) and how the editor was applied.*

Authentication: *Describe any authentication procedures for each seed stock used or novel genotype generated. Describe any experiments used to assess the effect of a mutation and, where applicable, how potential secondary effects (e.g. second site T-DNA insertions, mosiacism, off-target gene editing) were examined.*

Flow Cytometry

Plots

Confirm that:

- ☒ The axis labels state the marker and fluorochrome used (e.g. CD4-FITC).
- ☒ The axis scales are clearly visible. Include numbers along axes only for bottom left plot of group (a 'group' is an analysis of identical markers).
- ☒ All plots are contour plots with outliers or pseudocolor plots.
- ☒ A numerical value for number of cells or percentage (with statistics) is provided.

Methodology

Sample preparation: Sample preparation methods are described in Methods section of the manuscript. Typically, cells were incubated with

fluorescent reagents for 20 min on ice, before washing and data acquisition using the cytometer.

Instrument	4-laser BD LSRFortessa X-20 (BD Biosciences); 5-laser Cytek Aurora (Cytek Inc.)
Software	Data acquisition: BD FACSDiva 8.0.1 and Spectroflo 3.2.1; Data Analysis: FCS Express 4 and FloJo version 9.
Cell population abundance	Flow sorting was performed for: i) Generating single-cell isogenic clones. These were validated using Next-Generation Sequencing and based on lectin binding profiles. ii) Library screening. Enriched populations were assessed based on alterations in lectin binding profiles and cell-surface Fc expression. Following NGS sequencing, selected lectins were expressed independently and revalidated using independent assays.
Gating strategy	FSC/SSC was set for removing cell debris for all samples. Lymphocytes, granulocytes, and monocytes were gated using FSC/SSC and further analyzed. Subgating strategy was performed according to Cytek manual, and a representative figure for peripheral blood will be provided in complete manuscript.<br>FSC-A/FSC-H gates were additionally set to gate on single cells in cell line studies. Representative figures are present in current version of the manuscript.

☒ Tick this box to confirm that a figure exemplifying the gating strategy is provided in the Supplementary Information.
